# A Disposable and Multi-Chamber Film-Based PCR Chip for Detection of Foodborne Pathogen

**DOI:** 10.3390/s18093158

**Published:** 2018-09-19

**Authors:** Nam Ho Bae, Sun Young Lim, Younseong Song, Soon Woo Jeong, Seol Yi Shin, Yong Tae Kim, Tae Jae Lee, Kyoung G. Lee, Seok Jae Lee, Yong-Jun Oh, Yoo Min Park

**Affiliations:** 1 Department of Materials Science and Engineering, Hanbat National University, Daejeon 34158, Korea; nhbae@nnfc.re.kr; 2 Nano-Bio Application Team, National NanoFab Center (NNFC), 291 Deahak-ro, Yuseong-gu, Daejeon 34141, Korea; sylim@nnfc.re.kr (S.Y.L.); yssong@nnfc.re.kr (Y.S.); swjeong@nnfc.re.kr (S.W.J.); syshin@nnfc.re.kr (S.Y.S.); tjlee@nnfc.re.kr (T.J.L.); kglee@nnfc.re.kr (K.G.L.); sjlee@nnfc.re.kr (S.J.L.); 3 Department of Chemical Engineering & Biotechnology, Korea Polytechnic University, 237 Sangidaehak-ro, Siheung-si 15073, Gyeonggi-do, Korea; ytkim@kpu.ac.kr

**Keywords:** film PCR chip, PCR, pathogen DNA amplification, point-of-care testing

## Abstract

Since the increment of the threat to public health caused by foodborne pathogens, researches have been widely studied on developing the miniaturized detection system for the on-site pathogen detection. In the study, we focused on the development of portable, robust, and disposable film-based polymerase chain reaction (PCR) chip containing a multiplex chamber for simultaneous gene amplification. In order to simply fabricate and operate a film-based PCR chip, different kinds of PCR chambers were designed and fabricated using polyethylene terephthalate (PET) and polyvinyl chloride (PVC) adhesive film, in comparison with commercial PCR, which employs a stereotyped system at a bench-top scale. No reagent leakage was confirmed during the PCR thermal cycling using the film PCR chip, which indicates that the film PCR chip is structurally stable for rapid heat cycling for DNA amplification. Owing to use of the thin film to fabricate the PCR chip, we are able to realize fast thermal transfer from the heat block that leads to short PCR amplification time. Moreover, using the film PCR chip, we could even amplify the target pathogen with 10 CFU mL^−1^. The artificially infected milk with various concentration of *Bacillus cereus* was successfully amplified on a single film PCR chip. On the basis of the reliable results, the developed film PCR chip could be a useful tool as a POCT device to detect foodborne pathogens via genetic analysis.

## 1. Introduction

The interest in point-of-care testing (POCT) with portable, inexpensive, and disposable devices is dramatically growing because of its potential applications in human healthcare and medical diagnostics [1,2,3,4]. Among the various applications, the molecular diagnosis with the assistance of polymerase chain reaction (PCR) has been considered to be one of the most important tools [5]. The PCR enables effective and selective target identification via rapid amplification even with a few copies of the gene [6].

Most of the conventional bench-top PCR machines operate in the temperature ranges from four to 95 °C with 40 cycles, but they show slow heating and cooling rates due to their high thermal mass. To overcome, the time consuming thermal cycling process driven by the conventional system, microfluidic-based lab-on-chip techniques aim to achieve enhancement of heat and mass transfer, decreases to the reagent volume, and precision control of PCR condition [7]. Up-to-date, various PCR devices have been fabricated with silicone, glass, plastic, and hybrid of silicon, glass, and plastic [8,9,10,11,12]. While currently developed microfluidic-based PCR devices have successfully demonstrated its performance, the silicone and glass materials are still suffering from some drawbacks such as PCR inhibition of untreated surface, optical opacity, high processing cost, and relatively low thermal conduction. Recently, polymeric materials such as cyclic olefin copolymer (COC), polydimethylsiloxane (PDMS), poly(methyl methacrylate) (PMMA), and polycarbonate has become an alternative choice with low cost, easy fabrication and mass production, optical transparency, and biocompatibility [13,14,15,16]. However, these polymers have poor thermal conductivity and limits to the rapid thermal response.

Lesson learned from the previous studies indicated that the advanced PCR device should: (1) Be low cost, compact, and portable for easy use; (2) be transparent and biocompatible; and (3) be improved in thermal conductivity and response. Herein, we report on a disposable, reliable, and thin polyethylene terephthalate (PET) film-based PCR chip with the assistance of a plotting cutter. The thin film allows improvement of thermal response and direct printing of PCR chamber design has the potential advantages for simplifying design and fabrication processes, PCR reagent loading steps, and recovery of the PCR product. To gain the insight of the mechanical stability of the chip under PCR processes, the leakage test was also carried out and visually confirmed. Additionally, the heat flow profiles also investigate under the different dimension of the PCR chip by varying the width of the channels. Moreover, to evaluate the PCR performance, the foodborne pathogen of *Bacillus cereus* was selected as an example model to confirm the performance of film-based PCR chip [17,18]. To accomplish the gene amplification using the developed PCR chip, the *Bacillus cereus*, which is the one of a significant foodborne disease causal pathogen, was selected as a target pathogen for food poisoning diagnosis [19,20,21]. To amplify the *Bacillus cereus* using the film PCR chip, the genomic DNA (gDNA) was extracted from the pathogen and mixed with PCR mixture, containing the polymerase, dNTPs, and primers. Then, the cocktail solution was injected into the inlet on the top layer of the PCR chip. The prepared chip was then located onto the national instrument (NI) device, which was specifically customized to provide the heat to the film PCR chip. Then, the amplified gene was measured by conventional gel electrophoresis method. By using the designed film PCR, the 1.0 × 10^1^ colony form of the unit (CFU) mL^−1^ of *Bacillus cereus* was sensitively evaluated. Therefore, we assume that our PCR chip could effectively and successfully evaluate the low concentration of the target pathogen, and the developed chip could be suitable for practical diagnosis as a point-of-care testing (POCT) device.

## 2. Materials and Methods

### 2.1. Materials and Instruments

GoTaq^®^DNA polymerase (M3005) was purchased from Promega (Madison, WI, USA). *Bacillus cereus* (ATCC 21768) were purchased from ATCC. 2′-(4-Hydroxyphenyl)-5-(4-methyl-1-piperazinyl)-2,5′-bi-1H-benzimidazole trihydrochloride hydrate and 2′-(4-hydroxyphenyl)-5-(4-methyl-1-piperazinyl)-2,5′-bi-1H-benzimidazole trihydrochloride hydrate, bisBenzimide (HEPES) were obtained from Sigma-Aldrich. (St. Louis, MO, USA) Go Taq^®^ DNA polymerase (M3005) was purchased from Promega. NI PXIe-1060Q (NI) was obtained from National Instruments. QuickExtract™ DNA extraction solution 1.0 (QE09050) was obtained from Epicentre. The thermal cycler (C1000Touch™) was obtained from Bio-Rad (Hercules, CA, USA). LIAS Slite 140 was purchased from Avegene Life Sciences. The plotting cutter (FC4600C-50 PRO) was obtained from GRAPHTEC (Tokyo, Japan).

### 2.2. Fabrication of Film-Based PCR Chip

The three different film PCR chips were designed using AutoCAD (Autodesk, San Rafael, CA, USA) and further fabricated using plotting cutter (FC4600C-50 PRO). The PCR chip consists of three polymer films with five of PCR chambers, inlet, and outlet holes, respectively. The PET film was employed to the top and bottom layers by sequentially stacking the films. The microfluidic chamber between the top and bottom layers were manipulated by cutting PVC double-side adhesive film via plotting cutter. The one single PCR chip contains the five individual PCR chamber, and each PCR chamber contains the two-hole for inlet and outlet on the top layer. By alternately stacking each prepared film, the fabrication of film PCR chip was completed and the final volume of each chamber was 20 µL.

### 2.3. Preparation of Bacillus cereus Based on Broth and Milk

The standard Luria-Bertani (LB) broth including the 1 g of NaCl, 0.5 g of yeast extract, and 1 g of tryptone in 100 mL of autoclaved DI water was employed for culturing the *Bacillus cereus* under the temperature at 37 °C for 18 h. The number of cells suspended in the broth culture was calculated by the colony counting method [22]. The various concentration of *Bacillus cereus* was prepared by spiking the suspended pathogens into a broth or milk with serial dilution in the concentration range from 10^1^ to 10^5^ CFU per 10 µL of broth or milk. The prepared samples were stored at 4 °C until use up to a week.

### 2.4. Forward and Reverse Primers for Bacillus cereus

The groEL gene of genomic sequences of *Bacillus cereus* was selected as target genes obtained from GenBank. For amplification of the gDNA of *Bacillus cereus*, the 5′-GCTGGTGCGAACCCAATG-3′ and 5′-TCGCCTTCTACATCTTCAGCA-3′ was synthesized for forward and reverse primers. The amplicon size of the target gene was 443 for *Bacillus cereus*. The primers were modified to enhance the gene amplification efficiency for proper application regarding the previous research [23].

### 2.5. Optimization of Film PCR Chip

The various type of film PCR was optimized to verify the heat transfer efficiency. The 6 × 3 mm with 920 µm, 12 × 3 mm with 460 µm, and 18 × 4 mm with 230 µm film PCR chips were designed and fabricated. The heat transfer simulation of each designed film PCR chip was conducted by COMSOL software. Those film PCR chips were also fabricated as designed, and the *Bacillus cereus* gene amplification test was implemented.

### 2.6. Bacillus cereus Gene Amplification Using the Film PCR Chip

The gDNA was extracted by using the QuickExtract^TM^ solution as the following steps: (1) Preparation of 1.0 × 10^8^ CFU of *Bacillus cereus* cell; (2) centrifugation of the cells to obtain the pellet; and (3) addition of 100 µL extraction solution with 98 °C incubation for 2 min. The various concentrations of *Bacillus cereus* samples were prepared by serially diluting the lysed cells into the extraction solution. The extracted gDNA solution was pre-mixed with the PCR cocktail including the final concentration of 0.8 mM dNTPs, 1.5 mM MgSO_4_, 0.8 µM primers, and 6.25 U polymerase. The prepared PCR solution containing 19.2 µL PCR mixture and 0.8 µL DNA template was then manually loaded into the film PCR chip using a pipette, and the PCR chip was applied to the NI device. The NI device contains the copper heater with a cooling fan, which is controlled by software, and thus the thermal cycling is possible by adjusting the thermal algorithm in the control program. The heater size is the 3.5 × 3.5 cm. The thermal cycling was implemented under the following condition: Pre-denaturation at 95 °C for 300 s, denaturation of DNA template at 95 °C for 30 s, annealing of primer at 60 °C for 30 s, synthesizing the DNA at 72 °C for 30 s, and elongation at 72 °C for 300 s. The thermal cycle is repeated as 30 times. After completion of gene amplification using the film PCR chip, the amplified gene was manually recovered and visualized by the gel electrophoresis method.

## 3. Results

### 3.1. Fabrication of Film PCR Chip

**Figure 1:**
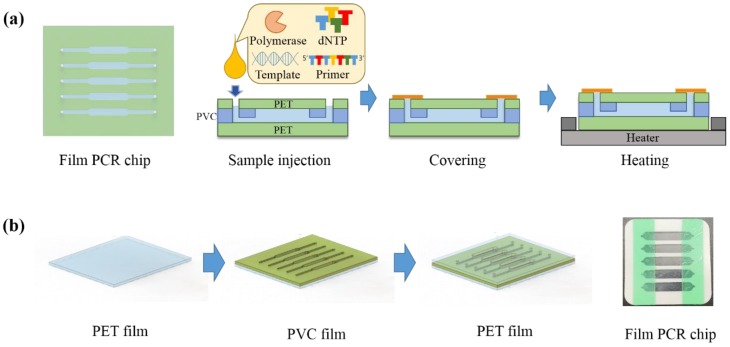
(**a**) Schematic illustration of gene amplification by using the film polymerase chain reaction (PCR) chip. The film PCR chip could effectively amplify the pathogen gene by applying to the minimized heating device; and (**b**) The simple designed multiplex film PCR chip, which consist of polyethylene terephthalate (PET) and PVC was fabricated by stacking each prepared film layer by layer.

To achieve the demonstration of thin film microfluidic-based minimized PCR chip, the polyvinyl chloride (PVC) and polyester (PET) films were employed in the study. To simultaneously amplify the gene from target samples in a single chip, the five individual microfluidic channels were fabricated on one chip, as shown in Figure 1a. The film PCR chip consists of three polymeric film layers. The PET film was employed as the top and bottom layer to support and cover the middle layer. The double-sided adhesive PVC film was used as the middle layer for the construction of PCR chambers. The top layer also contains inlet and outlet holes in accordance with each PCR chamber to inject the various solutions. Each film was modified in accordance with its own functionalities, and the fabrication of film PCR chip was utilized by sequentially stacking each prepared films.

Figure 1b illustrate the fabrication steps of the film PCR chip. To utilize the film PCR chip, the various films such as polyethylene terephthalate (PET) and polyvinyl chloride (PVC) were designed and directly printed using plotting cutter. The film PCR chip was manipulated by stacking each designed film in layer by layer. At first, the flat and a thickness of 100 μm PET film is prepared and the PCR chamber embedded double side adhesive PVC film with a thickness of 230 μm, which was carefully attached on the top of bare PET film. The double-adhesive PVC film contained five individual chambers for simultaneous gene amplification on a single chip. Finally, the top of these assembled chips were covered with PET film that have five inlet holes and five outlet holes to inject and recover PCR reagent and PCR product, respectively. Overall, the total size of the film PCR chip is the 3 cm × 3 cm, and the volume of each chamber is 20 µL.

### 3.2. Understanding of Temperature Profiling and Heat Stability of Film PCR Chip

**Figure 2:**
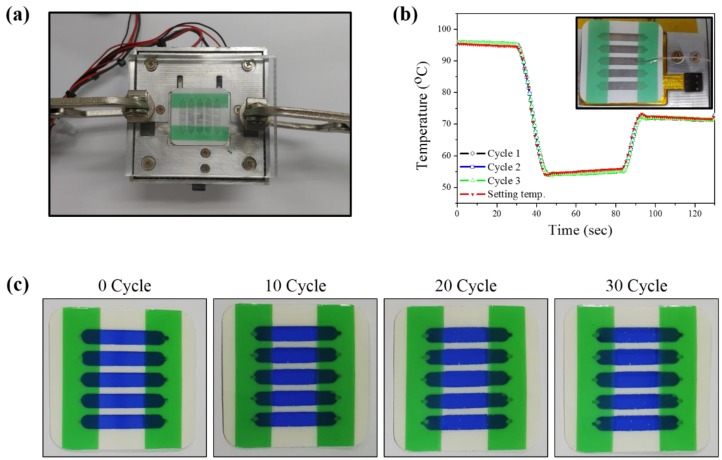
(**a**) The film PCR on the NI instrument for thermal cycling. The acryl cover with tong was employed for effective heat transfer and preventing the solution leakage; (**b**) the thermal profiling in the three cycle. The temperature in the PCR chamber was highly matched in all tested cycle with setting temperature; and (**c**) the leakage test in various thermal cycle step using the blue ink. The result images were recorded in every 10 thermal cycle, and the leakage was not found in all test result.

The heat transfer efficiency is directly related to the gene amplification, and thus, the temperature-profiling test was implemented in order to verify the heat transfer of the solution in the chamber [24,25,26,27]. The Figure 2a shows the thermal cycling system. The solution-injected film PCR chip was located onto the thermal cycling system that is controlled by national instrument (NI) equipment, and the thick acryl plate covered the top of film PCR chip to realize strong and evenly contacted the chip on the heater during PCR. To investigate the heat profiles inside of film PCR, the thermocouple is inserted in the chamber in the PCR chip, and the heat profiling of the input and output thermal cycling was monitored. The test result of repeated heat cycle is presented in Figure 2b. In the red line, the standard thermal cycling for PCR was entered, and then the output data was overlapped on the input graph. The result data from output value in the whole thermal cycling range was significantly matched with input data without deviation, indicating that the employed films could stably transfer the heat to the internal solution. While the overshooting and undershooting behavior was observed in every heat alteration point, the temperature was rapidly reached to the target level less than one s. Additionally, these phenomena were uniformly registered in the repeated thermal cycling. Based on the findings, we validated that the designed and fabricated film PCR chip could effectively deliver the heat to the solution and precisely controlled by NI equipment.

The employed film PCR chip contains total 10 of the inlet and outlet holes for PCR reagent and recovers of PCR product and the holes should be securely enclosed to avoid the potential leakage during the thermal cycling that directly involves PCR efficiency. To implement the leakage test, the blue ink was loaded into individual PCR chambers, and the adhesive PET film was attached to seal the holes. The solution leakages were observed every 10 thermal cycling using as-prepared film PCR device. Each cycle operates that is operated from 4 to 95 °C. After 10 thermal cycling test, as shown in Figure 2c, there was no visible leakage and/or evaporation of the blue ink. Then, we extended the thermal cycling test up to 30 cycles. In the result, the photographic images of Figure 2c from 20 and 30 times thermal cycled film PCR chip, no visible leakage and/or evaporation of ink were found, as compared to the initial status of ink inside of film PCR chip. These experimental results indicated that the employed PET film and PCR chip surely protected the solution from both inside and outside environment. Based on the findings, the thermal cycling could be stably implemented by using the developed film PCR chip.

### 3.3. Optimization of Film PCR Chip Type Using the Heat Transfer Simulation

**Figure 3:**
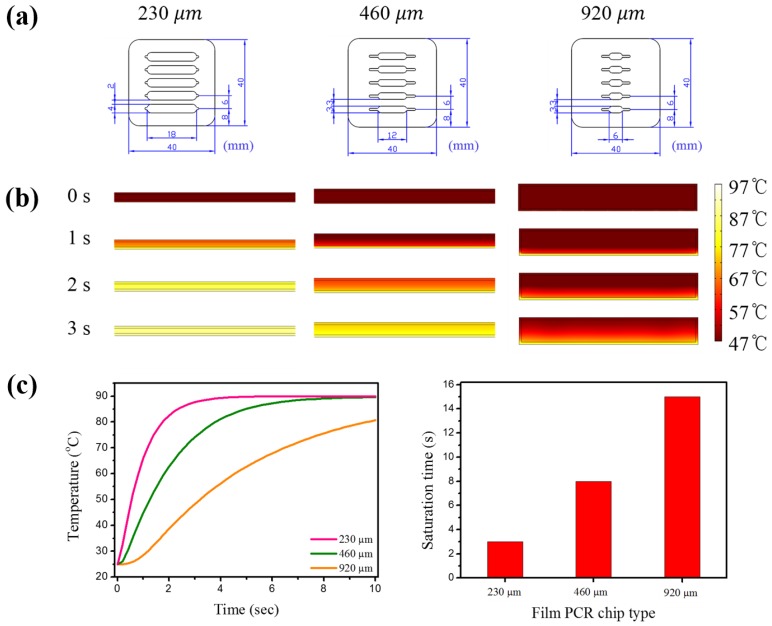
(**a**) The dimension of three type designed film PCR chip which contains the various width, length, and height (18 × 4 mm with 230 µm, 12 × 3 mm with 460 µm, and 6 × 3 mm with 920 µm) with same total volume of 20 µL; (**b**) the heat transfer simulation for 3 s in each designed film PCR chip by COMSOL software; and (**c**) the calibration curve for temperature and saturation time in each film PCR chip.

To successfully conduct the thermal cycling PCR, the heat transfer rate is significantly crucial to determine the PCR efficiency. To optimize the heat efficiency, the type of film PCR chips were designed and compared, as shown in Figure 3a. The fabricated film PCR has the same chamber volume as the 20 µL, while the length, breadth, and height are various to figure out the structural relationship between the heats transmit distances with transfer efficiency. The 6 × 3 mm with 920 µm, 12 × 3 mm with 460 µm, and 18 × 4 mm with 230 µm were designed, and the height was adjusted by the thickness of double-sided adhesive PVC film. Employing the COMOSL program involved with heat transfer simulation [28,29], the heat migration in the chamber from each film PCR chip was registered from zero to three s.

Figure 3b shows the thermal transfer rate in the chamber at the top area. In the results from 230 µm chip, the heat could be rapidly reached to the high temperature within a few seconds, while the 460 and 920 µm chip demonstrated relatively low heat transfer. The reason for the results was that the 230 µm PCR chip had the wide contact surface area allowing the effective heat transfer, while the 460 and 920 µm has a relatively narrow surface area inducing the decline of efficiency. To verify the heat correlation with each PCR chip, the 10 s heat profiling was presented in the Figure 3c (left). First, the 230 µm chip was reached to the target temperature, and the 460 and 920 µm chips were sequentially increased. In the Figure 3c (right), the saturation time of each chip was three, eight, and 15 s for 230, 460, and 920 µm chips, respectively. As the individual thermal cycle step could be conducted for 30 s, the rapid thermal transfer is significant for PCR efficiency. Based on the obtained test results, the 230 µm PCR chip exhibited the high performance, and the optimized chip was applied to the pathogen evaluation.

### 3.4. Performance Evaluation of Film PCR Chip via Pathogen Gene Amplification

**Figure 4:**
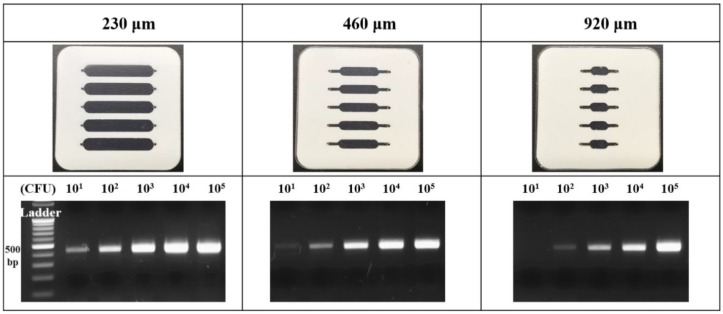
The fabricated three type film PCR chip as designed and optical images for *Bacillus cereus* gene amplification result. The 1.0 × 10^1^ to 1.0 × 10^5^ CFU mL^−1^ *Bacillus cereus* was evaluated.

The small amount of *Bacillus cereus* causes the several infectious diseases involved with foodborne illnesses. The bacteria lead to 73,000 illnesses, 2200 hospitalizations, and 60 deaths annually in the United States estimated by the Centers for Disease Control and Prevention (CDC), and *Bacillus cereus* is the significant foodborne disease causal pathogen [19,20,21]. To amplify the target gene, the prepared pathogen was lysed and purified, and the gene and PCR reagents were manually injected into the inlet. The hole of the inlet and outlet is secured to prevent the solution leakage and air inflow. Then, the film chip is thermal cycled using the NI equipment. After the film PCR, the amplified gene was evaluated gel electrophoresis method. Based on the fabricated film PCR chip, the target pathogen gene could be easily amplified, and the details are described in the following section. To implement the multiple pathogen gene amplification on a single chip, various concentrations of target pathogen were prepared and applied. As the small amount (less than 10 CFU mL^−1^) of bacteria in the food could have caused the food poisoning, we focused on the evaluation of low concentration pathogen. The *Bacillus cereus* 1.0 × 10^1^ to 1.0 × 10^5^ CFU mL^−1^ were prepared and tested using the fabricated film PCR chip. To accurately compare the gene amplification efficiency according to the chip type, the three types of PCR chips were fabricated as shown in the Figure 4.

The 20 μL sample solution containing a various concentration of 2 μL *Bacillus cereus* extract was separately injected into each inlet hole, and the hole on the PCR chip was precisely covered by PET film. The prepared chip was located onto the NI instruments, and the 30 thermal cycles was performed. To verify the productivity of film PCR chip, 3 μL PCR product in the chamber was confirmed by the gel electrophoresis analysis, as shown in Figure 4 (bottom). In the 230 μm chip, the PCR bands intensity was changed in proportion to the applied *Bacillus cereus* concentration with a clear observation in the whole detection range. Thus, the limit of detection (LOD) of the 230 μm chip to the *Bacillus cereus* evaluation is 1.0 × 10^1^ CFU mL^−1^. In the 460 μm chip, the band was detected from 1.0 × 10^2^ to 1.0 × 10^5^ CFU mL^−1^ of *Bacillus cereus*, and the 1.0 × 10^1^ mL^−1^ was faintly observed, exhibiting the LOD as 1.0 × 10^1^ CFU mL^−1^. The 920 μm chip demonstrated that the relatively low optical signal in the whole test range, and the chip could not effectively amplify the low concentration of the target gene from 1.0 × 10^1^ CFU mL^−1^. The LOD is the 1.0 × 10^2^ CFU mL^−1^ owing to the observation of light-colored PCR band. In regard to the obtained results, we verified that the 230 μm PCR chip with wide contact area shows the practically high performance to the gene amplification as well as effective heat transfer. The test result with reliable findings validated our success in establishing a useful molecular diagnostic device and designed PCR chip with gene amplification principle that significantly reduced the device size and cost for a single assay.

### 3.5. Detection of the Foodborne Pathogen in Real Samples

**Figure 5:**
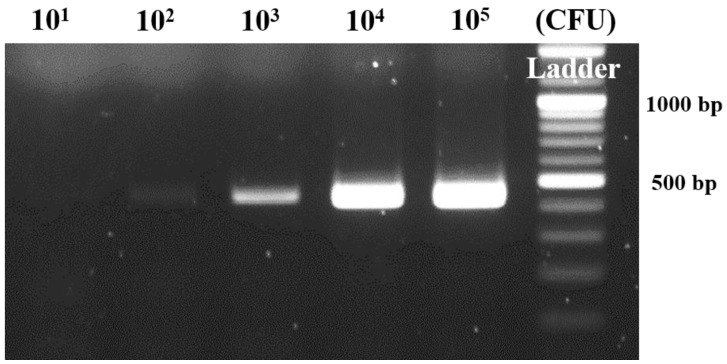
Milk-based *Bacillus cereus* gene amplification using the optimized condition with film PCR chip. The 1.0 × 10^1^ to 1.0 × 10^5^ CFU mL^−1^ *Bacillus cereus* was spiked and serially diluted in the milk.

To verify the practical usability of the developed film PCR chip, the real sample-based pathogen gene amplification was implemented. By using the multiplex chamber in film PCR chip, the various concentration of pathogen gene in the real samples were simultaneously amplified on a single chip. The 1.0 × 10^1^ to 1.0 × 10^5^ CFU mL^−1^ *Bacillus cereus* was serially diluted in the milk for the preparation of artificially infected food samples. The pathogen-spiked real samples were treated under the same condition with previous methods, and the milk lysates were loaded into the PCR chip, applying the NI instrument with the thermal cycle. The amplified gene was also evaluated by gel electrophoresis principle presented in the Figure 5.

While the PCR band was gradually increased in regard to the applied pathogen concentration in whole detection range, the relatively low optical intensity from the result images was measured in comparison with that of the previous test. The reason for the result was probably that the ingredients in the milk solution such as casein, albumin, and various proteins could interfere with gene amplification in terms of non-specific binding of ingredients to the chip surface or steric hindrance to the polymerase activity. Based on these phenomena, the 1.0 × 10^1^ CFU mL^−1^ was not observed, and the 1.0 × 10^2^ CFU mL^−1^ was ambiguously detected, indicating the LOD as the 1.0 × 10^2^ CFU mL^−1^. In regard to the requirement of minimization of gene amplification device in terms of lab-on-a-chip for the evaluation of pathogens causing the food poisoning, these approaches are significant for simplifying the molecular diagnostic system as a point-of-care testing device. To fulfill the demands of the clinical and commercial field for the fully integrated evaluation device, the research for film chips with various functionalities is undergoing.

## 4. Conclusions

In this study, we presented the novel fabrication of film PCR chip and its successful demonstration in performing the gene amplification and detection of the foodborne pathogen. The heat profile and computer simulation also carried out to investigate and confirm the distinctive environment for enhancing the heat and mass transfer via adopting thin polymer film. The surface areas to contact the heater and chamber design of the PCR chamber are critically important to increase the LOD of the genetic analysis. Among the three different kinds of PCR chamber, the PCR chip named of 230 μm showed excellent PCR performance and the detection limit was achieved down to 1.0 × 10^1^ CFU mL^−1^. Moreover, this film PCR would be applicable for rapid and on-site detection of foodborne pathogens and have a great potential towards POCT applications.
